# Time‐resolved fluorescent microsphere lateral flow biosensors for rapid detection of *Candidatus* Liberibacter asiaticus

**DOI:** 10.1111/pbi.13828

**Published:** 2022-05-12

**Authors:** Cong Su, Fang Ding, Wenjing Wang, Zhiyong Song, Qurban Ali, Mubassir Ali, Ni Hong, Guoping Wang, Heyou Han

**Affiliations:** ^1^ State Key Laboratory of Agricultural Microbiology College of Science Huazhong Agricultural University Wuhan China; ^2^ Hubei Key Laboratory of Plant Pathology College of Plant Science and Technology Huazhong Agricultural University Wuhan China

**Keywords:** *Candidatus* Liberibacter asiaticus, Citrus, detection, Huanglongbing, time‐resolved fluorescent microsphere lateral flow biosensors

Citrus Huanglongbing (HLB) is one of the most devastating diseases causing unprecedented global economic losses and constituting a major hindrance to the development of the citrus industry. ‘*Ca*. Liberibacter asiaticus’ (*C*Las) is the most prevalent strain. Almost all commercial citrus cultivars are sensitive to it, and no curable method is available. Accurate plant disease diagnoses and rapid detection of plant pathogens are of utmost importance for appropriate application of phytosanitary measures (De Boer and Lopez, 2012). Currently, accurate, timely and robust detection of *C*Las is still challenging. Lateral flow biosensors (LFBs) are paper‐based devices, which is a leading technology for point‐of‐care testing of pathogens with the advantages of portability, on‐site testing, rapidity, low cost and no need of professional operators (Quesada‐Gonzalez and Merkoci, 2015). The fluorescent microsphere (FM), nanometer‐to‐micron particles with advantages of stable configuration, high‐fluorescence intensity and photostability, has become a novel fluorescent label and is widely used as probe (Wang *et al*., 2020). As compared to conventional colloidal gold nanoparticles, fluorescent microprobes showed greatly improved analytical sensitivity (Yang *et al*., 2021). Furthermore, time‐resolved fluorescent material endowed the LFBs with high anti‐interference capability against intrinsic background fluorescence from complex biological samples, thus improving the performance of classical biosensors (Lee *et al*., 2020). Although a variety of methods have been developed for the identification of *C*Las, a user‐friendly, rapid and accurate on‐site testing is still in an urgent need. In our previous work, specific polyclonal and monoclonal antibodies against *C*Las were obtained (Ding *et al*., 2020), which were used for the development of biosensors. In the current research, a novel time‐resolved fluorescent microsphere lateral flow biosensors (TRFM‐LFBs) for the detection of *C*Las in a quantitative manner with high sensitivity, selectivity and rapidity was developed. The working flow was described in Figure 1a.

**Figure 1:**
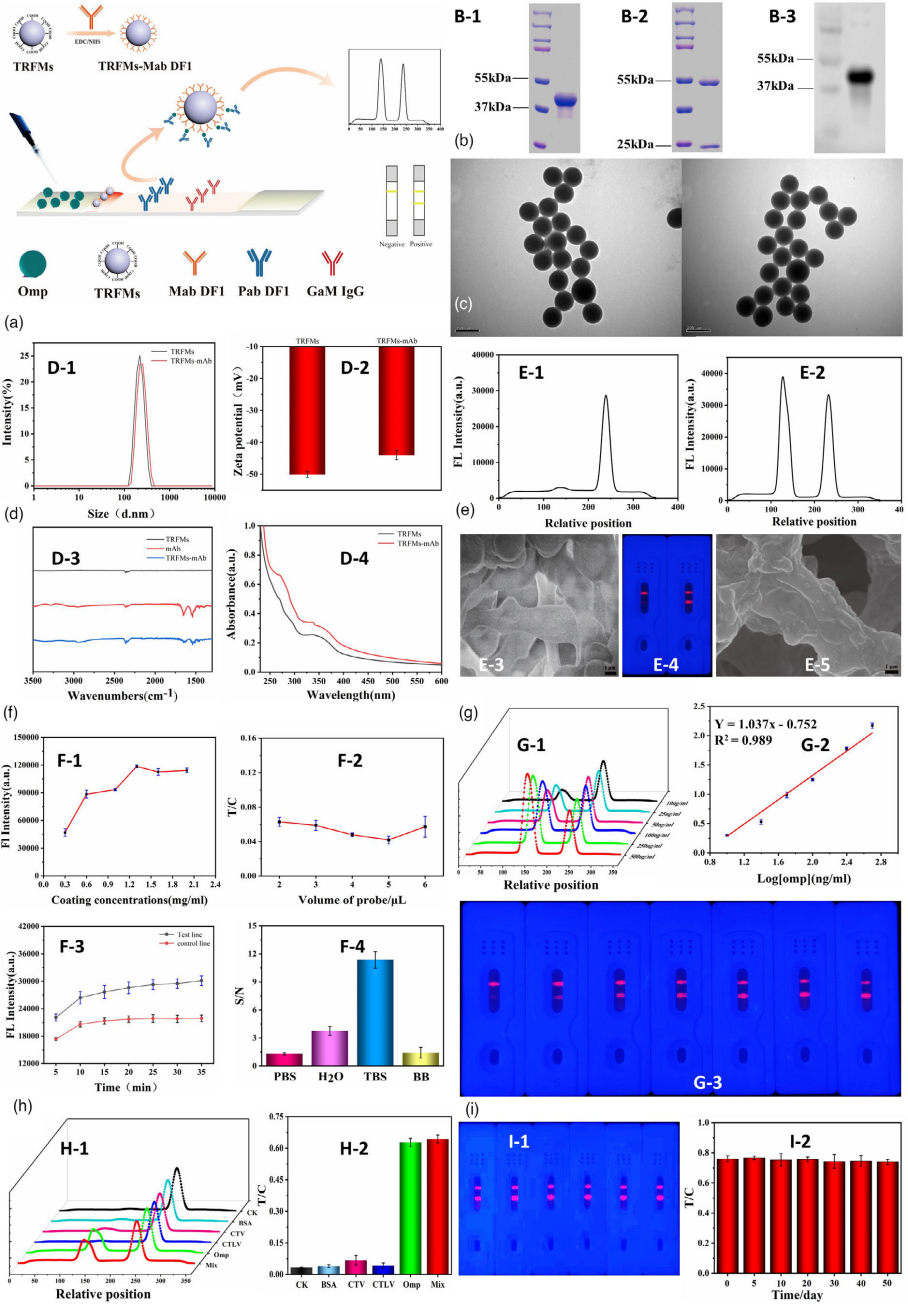
Time‐resolved fluorescent microsphere lateral flow biosensors for rapid detection of *C*Las. (a) Schematic illustration of time‐resolved fluorescent microsphere lateral flow biosensors (TRFM‐LFBs) for the detection of *C*Las. Expression and characterization of purified Omp A protein (43KD) proteins through NI‐NTA affinity chromatography (b‐1) and the Mab DF1 (b‐2). Western blot analysis of purified Omp A proteins using Mab DF1 antiserum (b‐3). (c) TEM observation of TRFMs before (c‐1) and after (c‐2) modification with MabDF1 antibody. (d) TRFMs‐Mab DF1 conjugates verified by dynamic light scattering (d‐1), Zeta potential (d‐2), IR spectra (d‐3) and UV‐Vis spectra (d‐4) analysis. (e) Feasibility of the TR‐LFB method: absence (e‐1, e‐3 and e‐4 left) and presence (e‐2, e‐4 right and e‐5) of Omp A protein. (f) Optimization of the main factors: concentration of GaM IgG (f‐1), TRFMs‐Mab DF1 (f‐2), the incubation time (f‐3) and the reaction buffers (f‐4). (g) TRFM‐LFBs strip for assaying *C*Las at different concentrations. The detection limitation was 6.88 ng/mL (S/N > 3) (g‐1, g‐3) with linear equation *Y* = 1.037*x*–0.752 (*R* ^2^ = 0.989) (g‐2). (h) Specificity assays of TRFM‐LFBs strip. No signal (h‐1, h‐2) was obtained except for *C*Las. (i) Stability analysis revealed fluorescent signals can be detected in 50 day storage under RT conditions (i‐1, i‐2).

Since the Omp A protein is conserved and widely distributed in all *C*Las strains (Ding *et al*., 2020); therefore, the monoclonal antibody specifically against Omp A (namely, Mab DF1), which was produced from cloned hybridoma cells (*C*Las J8B) was used. Mab DF1 was covalently conjugated with the carboxylate‐modified fluorescent microsphere (TRFMs‐Mab DF1), which is polystyrene (PS‐COOH, 1 wt%) in diameter of 210 nm, with the excitation of 365 nm and the emission of 610 nm (MD013, Microdetection, Nanjing, China). The TRFMs‐Mab DF1 conjugates were used as signal probes to trace *C*Las. The corresponding polyclonal antibody (Pab DF1) was produced by immunizing the rabbits with purified recombinant Omp A protein (Figure 1b) and immobilized as the test line on the LFB.

The fabrication of TRFMs‐Mab DF1 was following a protocol (You *et al*., 2019) and was subsequently characterized by transmission electron microscope (TEM). The shape of both TRFMs before and after conjugation with Mab DF1 was nearly round, and the size dispersity is uniform (Figure 1c). Further analysis of the dynamic light scattering (Figure 1d‐1), Zeta potential (Figure 1d‐2), IR spectra (Figure 1d‐3) and UV‐Vis spectra (Figure 1d‐4) revealed Mab DF1 was successfully conjugated with TRFMs. To fabricate the TR‐LFB biosensor, four parts of the sample pad, conjugate pad, NC membrane and adsorbent pad were assembled on a sheet of plastic adhesive backing. Each pad was overlapped with the adjacent one to ensure the fluent migration of loaded test samples. Pab DF1 and the secondary goat anti‐mouse IgG were sprayed onto the NC membrane as the T and C lines, respectively. The observation of the fluorescent signal is illuminated with the UV light at 365 nm. To verify the feasibility of the TR‐LFB method, a sandwich ELISA was used to capture the target Omp A protein. Theoretically, fluorescent signals on the control line should always be present, indicating the validation of TR‐LFB test. Meanwhile, the presence or absence of fluorescent signals on test line indicates the positive/negative results for the samples tested. As revealed by portable fluorescence reader and TEM, when the target Omp A protein was absent, no fluorescent signals (FSs) were tested (Figure 1e‐1 and e‐4) and no TRFMs‐Mab DF1 were captured. But, when Omp A protein was present, FSs and TRFMs‐Mab DF1 were both observed (Figure 1e‐2 and e‐5). All the main factors including the concentration of goat anti‐mouse (GaM) IgG in the C line (Figure 1f‐1), TRFMs‐Mab DF1 (Figure 1f‐2), the incubation time (Figure 1f‐3) and the reaction buffers (Figure 1f‐4) were optimized. An optional TRFM‐LFBs working assay was finally obtained: adding 5 μL of TRFMs‐Mab DF1 gave the best signal‐to‐background ratio. While 1.2 mg/mL of goat anti‐mouse IgG in the C line always produced stable signals. Comparison of 4 kinds of reaction buffer revealed TBS (pH = 8.0) led to the optimal response. In addition, 25 min was enough to get best results.

To assess the performance of the TRFM‐LFBs strip for the detection limitation of *C*Las. A series of dilutions of Omp A protein was tested (Figure 1g). Under the optimal conditions, the linear equation is *Y* = 1.037*x*–0.752 (*R* ^2^ = 0.989). The detection limit was determined to be 6.88 ng/mL (S/N > 3). Specificity assays revealed the TRFM‐LFBs strip specifically recognized *C*Las. All the other pathogens including CTV, CTLV, and blank control gave no signal (Figure 1h). Stable fluorescence intensity ratio of both T and C lines of TRFM‐LFBs strips can be detected in 50 d storage under RT conditions (Figure 1i).

In conclusion, we firstly established a novel time‐resolved fluorescent microsphere lateral flow biosensors (TRFM‐LFBs) for the detection of *C*Las. With the help of a portable FL strip reader, TRFM‐LFBs is a promising ideal point‐of‐care testing device for the rapid and accurate identification of *C*Las.

## Conflict of interest

The authors declare no conflicts of interest.

## Author contributions

C. Su and F. Ding initiated the study and contributed equally; H. Y. Han and G.P. Wang designed the experiment. Q. Ali and M. Ali prepared the samples. W.J. Wang, Z.Y. Song, Ni. Hong, F. Ding and H. Y. Han contributed to critically revising of the manuscript.
